# Infection-Associated Mechanisms of Neuro-Inflammation and Neuro-Immune Crosstalk in Chronic Respiratory Diseases

**DOI:** 10.3390/ijms22115699

**Published:** 2021-05-27

**Authors:** Belinda Camp, Sabine Stegemann-Koniszewski, Jens Schreiber

**Affiliations:** Experimental Pneumonology, Department of Pneumonology, University Hospital Magdeburg, Health Campus Immunology, Infectiology and Inflammation (GC-I³), Otto-von-Guericke University Magdeburg, 39120 Magdeburg, Germany; belinda.camp@med.ovgu.de (B.C.); jens.schreiber@med.ovgu.de (J.S.)

**Keywords:** asthma, COPD, neuro-immune interactions, neuro-inflammation, neuropeptides, rhinovirus, influenza A virus, respiratory syncytial virus, *Staphylococcus aureus*

## Abstract

Chronic obstructive airway diseases are characterized by airflow obstruction and airflow limitation as well as chronic airway inflammation. Especially bronchial asthma and chronic obstructive pulmonary disease (COPD) cause considerable morbidity and mortality worldwide, can be difficult to treat, and ultimately lack cures. While there are substantial knowledge gaps with respect to disease pathophysiology, our awareness of the role of neurological and neuro-immunological processes in the development of symptoms, the progression, and the outcome of these chronic obstructive respiratory diseases, is growing. Likewise, the role of pathogenic and colonizing microorganisms of the respiratory tract in the development and manifestation of asthma and COPD is increasingly appreciated. However, their role remains poorly understood with respect to the underlying mechanisms. Common bacteria and viruses causing respiratory infections and exacerbations of chronic obstructive respiratory diseases have also been implicated to affect the local neuro-immune crosstalk. In this review, we provide an overview of previously described neuro-immune interactions in asthma, COPD, and respiratory infections that support the hypothesis of a neuro-immunological component in the interplay between chronic obstructive respiratory diseases, respiratory infections, and respiratory microbial colonization.

## 1. Introduction

Chronic obstructive airway diseases such as chronic obstructive pulmonary disease (COPD) and bronchial asthma are generally characterized by airway inflammation leading to symptoms such as coughing, wheezing, and shortness of breath, and greatly affect the patient’s quality of life [1,2]. Depending on the disease, acute exacerbations occur due to the presence of allergens; pollutants; smoke; cold or dry air; and pathogenic bacteria, viruses or both [3,4]. Among these agents, viral infection is one of the major drivers of asthma exacerbations [5,6], and viral involvement in COPD exacerbations is equally high [7]. Rhinovirus (RV) and respiratory syncytial virus (RSV) are the predominant viruses linked to the development and exacerbation of chronic airway inflammatory diseases [8], but also other viruses like influenza A virus (IAV) have been implicated in acute exacerbations [9]. Respiratory viruses primarily infect and replicate within airway epithelial cells [10]. In chronically inflamed airways, antiviral responses can be impaired leading to sustained inflammation and erroneous infiltration of immune cells and cytokines, in turn resulting in the exacerbation of the underlying disease [11]. A variety of factors such as host genetic predisposition, age, and comorbidities contribute to a patient’s individual risk for exacerbations and altogether define the progression of chronic respiratory diseases. A role for infections and recently also for microbial colonization [12] of the respiratory tract in the onset and progression of chronic respiratory disease has also been appreciated [13], but the underlying mechanisms remain elusive in many points.

There is increasing evidence for neuro-immune crosstalk in inflammation; nevertheless, the role of infections orchestrating this crosstalk is still elusive. While our knowledge regarding neuro-inflammation in chronic respiratory diseases is increasing, also here there still are substantial gaps. These include the contribution to the initiation of disease as well as to the manifestation with respect to inflammatory phenotypes and the risk for exacerbations.

The respiratory tract receives somatosensory afferent innervation from neurons that reside within the dorsal root ganglion and vagal sensory innervation from neurons of the nodose/jugular ganglia [14]. Different neuropeptides secreted by neurons act on the vasculature and immune system, leading to immune cell recruitment and activation [15,16]. In allergic diseases, there is a direct correlation between the number of mast cells and neurons [17,18]. Furthermore, dendritic cells (DCs) are found closely associated to sensory neurons in the airways [19,20], and eosinophils, a key effector cell type in allergic reactions such as in allergic asthma, have been found to co-localize with cholinergic nerves [21].

In this review, we provide an overview of previously described neuro-immune interactions in the two main chronic respiratory diseases, asthma and chronic obstructive pulmonary disease (COPD). Furthermore, we discuss interactions along the neuro-immune axis as a potential mechanism of respiratory infections to contribute to the pathophysiology of chronic respiratory diseases. 

## 2. Chronic Respiratory Diseases

### 2.1. Bronchial Asthma

Bronchial asthma is one of the most common chronic respiratory diseases and is estimated to affect more than 300 million patients worldwide [22]. The symptoms of asthma vary over time as well as in intensity, and include wheezing, shortness of breath, chest tightness, and coughing [23]. The disease is characterized by bronchial hyper-reactivity (BHR), mucus overproduction, airway narrowing, and airway wall remodeling [24]. Remodeling processes involve an increase in airway smooth muscles, thickening of the subepithelial basement membrane, angiogenesis, neuronal proliferation, and metaplasia of goblet cells [25]. Asthma is an extremely heterogeneous disease and can be divided into non-allergic (intrinsic) and allergic (exogenous) asthma. Depending on the inflammatory endotype, the disease is divided into type 2-low and type-2-high asthma, the latter being the most common endotype [26]. Initial exposure to an allergen induces its uptake by antigen-presenting cells and finally results in an allergen-specific T helper type 2 (Th2)-response mainly mediated by T_H_2 cells, group 2 innate lymphoid cells (ILC2s), as well as IgE-producing B cells. This response typically includes the secretion of cytokines such as interleukin-4 (IL-4), IL-5, and IL-13 [22]. Re-exposure to the allergen results in degranulation of mast cells through binding to surface-bound IgE, leading to the release of histamine and other pro-inflammatory mediators that drive allergic inflammation [27,28]. Elevated IgE titers and eosinophilia characterize the disease [29,30,31]. Aiming at tailored measures for prevention and therapy, there is a large interest in defining the factors and mechanisms driving onset and chronic inflammation in allergic asthma and bronchial asthma in general. Over the last decades, there has been an increasing recognition that next to respiratory viral infections, also the presence of colonizing microorganisms in the upper airways has an impact [32]. Nevertheless, the interactions between microorganisms, chronic airway inflammation, and the involvement of neuro-inflammation are poorly understood.

### 2.2. Chronic Obstructive Pulmonary Disease

COPD is a major cause of death worldwide. It is characterized by not fully reversible airflow limitation, deregulated chronic inflammation, and emphysematous destruction of the lungs [33,34]. COPD generally manifests at an older age and the main risk factors are inhaled particles and gases from cigarette smoking and biomass fuel [35]. Nevertheless, there is also increasing evidence that early-life events have an impact on adult lung function [36]. Chronic inflammation in COPD involves the infiltration of inflammatory cells including neutrophils, macrophages/monocytes, lymphocytes, and in some patients also eosinophils into the airways and lung tissue. These infiltrations result in an excessive expression of proteases and inflammatory mediators that promote further accumulation of inflammatory cells. These processes also lead to remodeling of the airway structure [37]. As in asthma, there are highly heterogeneous inflammatory phenotypes in COPD. Also CD8^+^ T_c_2 cells have been shown to be significantly increased in the lungs of COPD patients where they mainly produce IL-4 and IL-5 [38] and asthma/COPD overlap syndromes are being discussed [39]. Comorbidities are ubiquitous among COPD patients and include cardiovascular diseases, osteoporosis, gastrointestinal diseases, and respiratory conditions such as asthma and lung cancer [40,41,42]. Due to its high heterogeneity and the progressive nature of the disease, effective therapy of COPD remains challenging and there is a high need for a better understanding of the different pathomechanisms at play.

## 3. Neuro-Immune Interactions in Allergic Asthma and COPD

### 3.1. Effects of Allergen Challenge on Sensory Nerves and Neuropeptide Release in Asthma

Research regarding neuro-immune crosstalk has focused increasingly on neuro-immune interactions in lung tissue and consequences for respiratory diseases like asthma and COPD, thereby highlighting its importance in this context. A number of immune cells share anatomical localization with peripheral neurons and crosstalk may have effects on inflammation [43]. Especially ILC2s were recently identified to play a key role in the induction of type 2 inflammation in the context of asthma [29]. The central nervous system (CNS) is the processing center of the human body and communication with peripheral tissues occurs via the peripheral nervous system (PNS). The brainstem can communicate with the lung via vagal sensory neurons, which are the fundamental units activated by sensory input from the environment [44]. Also, breathing is tightly regulated by the nervous system and several classes of central and peripheral sensory neurons regulate the respiratory cycle [45,46]. Sensory neurons of the vagus nerve are the major source of nerve fibers that innervate the lung and airways [45,47,48]. Lung nociceptors—a specialized subset of sensory neurons—can be stimulated by inhaled allergens such as house dust mites, but also viruses or bacteria, resulting in the calcium-mediated release of neuropeptides [49]. Neuropeptides such as substance P (SP) and calcitonin gene-related peptide (CGRP) generate neurogenic inflammation and modulate innate immune activation during infection [50]. Patients with asthma are hyper-responsive to SP, and SP causes bronchoconstriction, increases mucus secretion, and facilitates cholinergic neurotransmission and plasma leakage [51]. Previous investigations in mice showed that silencing nociceptor neurons reduced airway inflammation and bronchial hyper-responsiveness [49], possibly displaying a future therapeutic approach in allergic airway inflammation by interrupting sensory neuron-immune signaling. 

During allergic inflammation, sensory neuron-derived neuropeptides, including SP, vasoactive intestinal peptide (VIP), and neuromedin U (NMU), are released and directly affect immune cells. Vice versa, especially ILC2- and CD4^+^ T cell-derived IL-5 stimulates nociceptors to release VIP, which further enhances type 2 inflammation [49]. Further, it has been shown that the activation of ILC2s via NMU and the co-administration of NMU with IL-25 amplified allergic inflammation [52].

Mast cells often reside close to peripheral nerve endings where they release a broad range of pro-inflammatory cytokines and chemokines upon activation [53,54]. The neuropeptide SP facilitates immune cell migration and SP-mediated activation of human mast cells leads to the release of multiple pro-inflammatory cytokines via the homologous Mas-related G protein coupled receptor X2 (MRGPRX2) [53]. Recent advances have shown an interaction between this receptor with sensory neurons in the skin [55]. Additionally, its potential role in modulating allergic asthma is being increasingly recognized [56,57] and it now appears that human lung mast cells express MRGPRX2 [58]. Thus, it is possible that upon allergen challenge, SP is released from sensory nerves and activates MRGPRX2-expressing lung mast cells to mediate bronchoconstriction and Th2-mediated immune responses, resulting in mild allergic asthma. MRGPRX2 is upregulated in asthmatic lungs [57]. Therefore, it is likely that hemokinin-1 (HK-1) produced from lung macrophages, bronchial cells and mast cells [59,60], as well as ligands generated from eosinophils [61,62], neutrophils [63], and epithelial cells [64], further activate mast cells via MRGPRX2 to release inflammatory mediators and thereby amplify airway hyper-responsiveness, resulting in allergic asthma exacerbation [65]. Furthermore, Perner et al. revealed that allergen-induced SP release by transient receptor potential vanilloid (TRPV)^+^ sensory neurons triggers Mas-related G-protein coupled receptor member A1 (MRGPRA-1)-dependent DC migration to the lymph node, initiating the allergic immune response [66].

IL-31 regulates cell proliferation and is involved in tissue remodeling [67]. It also induces the production of inflammatory cytokines and chemokines in human keratinocytes, monocytes, and bronchial epithelial cells [68,69], suggesting that it may positively regulate inflammation in T2 diseases. Lai et al. show that serum and bronchoalveolar lavage (BAL) IL-31 levels were significantly elevated in patients with asthma, and IL-31 receptor alpha (IL-31 RA) mRNA was upregulated in OVA-challenged mice [70], indicating that IL-31 modulates the development of airway inflammation in asthma. Stimulation of dorsal root ganglia with IL-31 led to increased neuronal growth and neurite elongation [71], and IL-31 has been found to be significantly associated with total serum IgE levels, indicating a possible role in the development of asthmatic symptoms and their severity [72]. Currently, the anti-IL-31 receptor antibody Nemolizumab^TM^ is being clinically evaluated for the therapy of atopic dermatitis [73].

Regarding neuro-immune communication in the lung, also pulmonary neuroendocrine cells (PNECs) appear to influence T2 inflammation. PNECs are rare endoderm-derived airway epithelial cells that contain dense core vesicles filled with neuropeptides, amines, and neurotransmitters [74]. Their release of CGRP stimulates ILC2s and elicits downstream immune responses. They also induce goblet-cell hyperplasia via the neurotransmitter gamma-aminobutyric acid (GABA), and lungs from human asthmatics show increased PNECs [74].

Taken together, there are multiple interactions between neuronal regulation and T2 inflammation in allergic asthma. Their exact role and contribution to the disease need to be further clarified, possibly providing promising targets for future therapeutic strategies.

### 3.2. Neuro-Immune Crosstalk in COPD

While there is substantial knowledge reading neuro-immune interactions in allergic asthma, much less is known about the crosstalk between the nervous and the immune system in COPD. The disease is associated with shortness of breath, coughing, and bronchial obstruction. The majority of symptoms cannot only be attributed to immune cell infiltration, and there is evidence for a significant contribution of abnormal activity in bronchopulmonary vagal sensory neurons [75]. Nociceptive nerves—the majority of sensory nerves in the lung—are adept at sensing the type of tissue injury and inflammation that occurs in the lungs in COPD. These fibers are sensitive to decreases in pH and indeed the pH is likely to be decreased in COPD lungs at sites of tissue inflammation [76,77]. It also could be shown that patients with COPD show a reduction in sympathetic and parasympathetic activity [78]. The sympathetic nervous system controls bronchodilation and mucus production, whereas the parasympathetic nervous system controls bronchoconstriction [79].

VIP is one of the most abundantly expressed molecules in the human airways [80]; it relaxes pulmonary and coronary artery smooth muscles and has anti-inflammatory effects in animal models [81]. VIP also acts on bronchial mucus secretion [80]. Furthermore, studies show that VIP reduces cigarette smoke-induced damage of alveolar cells and apoptosis [81]. Two types of VIP receptors have been characterized, including vasoactive intestinal peptide receptors type I (VPAC1R) and vasoactive intestinal peptide receptors type II (VPAC2R). The expression of the VIP receptors VPAC1R and VPAC2R is increased in the central airways of smokers with chronic bronchitis [82]. This finding might suggest that regulation of mucus secretion by VIP is altered in COPD.

During airway inflammation, epithelial damage promotes reflective mechanisms that signal to both adaptive and innate immune cells by exposing vagal nerve endings in the submucosa to the airway lumen, leading to the release of acetylcholine (Ach) [14,83]. The release of Ach results in the activation of muscarinic 1 receptors (M1R) and muscarinic 3 receptors (M3R), which play a pro-inflammatory role in the lung [83,84].

Studies suggest a direct and indirect effect of SP in regulating neutrophil function and influx into inflamed tissues. Neutrophils may express SP, and SP enhances their phagocytic activity and regulates their influx into inflamed tissues [85]. Furthermore, the increase of neutrophil adhesion to bronchial epithelial cells mediated by SP [86] suggests an important role for SP in modulating neutrophilic airway inflammation. As COPD is predominantly characterized by neutrophilic inflammation [87], regulation of neutrophils by SP within this disease is likely. Neutrophils also play a prominent role in a subset of asthmatics. These neutrophilic asthma patients generally show increased disease severity, functional residual capacity, and exacerbations [88]. The regulation of neutrophils by SP could therefore potentially also play a key role in asthma types dominated by neutrophilic inflammation. 

In patients with COPD, an over-activation of PNECs compared to healthy subjects has been reported [89]. PNEC chemoreceptors and their secretion of peptides can cause increased chemoresponsiveness to odorants [89] and, therefore, also PNECS could display promising future therapeutic targets for managing COPD. Furthermore, in COPD patients, increased sputum CGRP levels [90] and increased numbers of CGRP-positive cells in the airways [89] have been reported.

While there are numerous implications for neurological involvements in COPD, especially neuro-immune crosstalk and its contribution to the disease need to be clarified in experimental and clinical studies in the future.

## 4. Neuro-Immune Crosstalk in Respiratory Infection and Microbial Colonization

There is evidence that viruses and bacteria that enter the respiratory tract or are part of the respiratory microbiome can directly activate nociceptors and therefore may have an impact on neuro-immune communication in chronic respiratory diseases [91]. They cause lung inflammation, irritation, and airway hyperreactivity (AHR), and also neuro-immune interactions occur during infection [79].

In general, infections cause the development and exacerbation of chronic airway inflammatory diseases [8,92]. It has long been recognized that respiratory viral infections trigger exacerbations of asthma in both children and adults, whereas for many years bacterial infection was thought to be the main trigger of exacerbations in COPD [93]. However, viral infections cause an increase in neutrophil activation, worsening symptoms and airway remodeling also in COPD [94]. The viruses and bacteria discussed below play a particularly important role in the context of asthma and COPD.

### 4.1. Infections with Human Rhinovirus

RVs belong to the most frequent viral pathogens in humans and are transmitted from person to person. RVs directly trigger the synthesis of inflammatory cytokines from epithelial cells, smooth muscle cells, and macrophages [95,96,97,98]. Little is known about how rhinoviruses affect the neuro-immune crosstalk in the respiratory tract, but there are studies showing RV infects neuronal cells [99]. RV infection causes upregulation of transient receptor potential (TRP) channels on sensory nerves while SP and toll-like receptor 4 (TLR4) RNA expression were reduced in infected children [99,100]. Subjects with RV infection showed an association between the expression of IL-1β, IL-6, and IL-8, and illness expression [101].

In the CNS, IL-1β is synthesized and released during injury, infection and disease, mediating inflammatory responses. However, IL-1β is also present in the brain under physiological conditions and can influence hippocampal neuronal function [102]. IL-6 is an essential regulatory factor for neuro-immunomodulation in the CNS, protecting neurons and promoting nerve regeneration [103,104]. The changes in IL-6 levels are strictly associated with neuropathological and physiological changes caused by inflammation or infection [105].

A key part of the response to viral infections is the production of interferons, which then activate their specific receptors. Type I interferons act directly on nociceptors [106]. Due to the potential of the cytokines released during infection to affect neurons, RV-infection possibly acts on the local nervous system with implications for any underlying chronic inflammatory respiratory disease.

### 4.2. Infections with Influenza A Virus

IAV is a highly contagious respiratory pathogen that remains a cause for morbidity and mortality throughout the world. There are several animal reservoirs for IAV, which, together with its genetic instability, constitutes its epidemic and pandemic potential [107,108]. It has been shown in a mouse model that in IAV infection the sympathetic nervous system increases pro-inflammatory cytokines and immune cell recruitment and thereby exacerbates morbidity and mortality [109]. Furthermore, persisting neuro-inflammation has been observed following infection with neurotropic as well as non-neurotropic IAV strains in mice [110]. Although lacking experimental or clinical proof, these findings imply IAV to potentially affect chronic respiratory diseases via modulation of the neuro-immune axis. 

Nerve- and airway-associated macrophages (NAMs) are a tissue-resident, self-renewing macrophage population in the lungs and are primarily localized around airways. They are also found close to the sympathetic nerves in the bronchovascular bundle. Ural et al. demonstrated that these cells are critical for regulating many aspects of the immune response and tissue homeostasis following inflammatory stimuli in the lung. During IAV infection in a mouse model, NAMs regulated virus-induced inflammation. They proliferated robustly following IAV infection, and in their absence, the inflammatory response was augmented, resulting in excessive production of inflammatory cytokines and innate immune cell infiltration [111]. Very recently, Verzele et al. showed that pulmonary IAV infection profoundly impacts the vagal sensory ganglia. Differential gene expression was accompanied by infiltration of MHC II^+^ cells into the vagal sensory ganglia of IAV-infected mice. Furthermore, the total number of CGRP immune-reactive sensory neurons and the number of inflammatory cellular infiltrates in the vagal sensory ganglia were significantly increased [112]. 

Furthermore, IAV infections are associated with strongly increased inflammatory gene expression in the respiratory tract [113]. The cytokines mainly released during IAV infection include type I and III interferons as well as pro-inflammatory cytokines such as tumor necrosis factor (TNF)-α, IL-1, and IL-6 [114]. While these cytokines on the one hand play a role in asthma and COPD, they potentially also lead to alterations in the local neuronal network. In this context, we have recently observed that IAV infection leads to sustained changes in lung homeostasis and significantly modulates subsequently induced allergic airway inflammation [115].

Altogether, these findings strongly support an influence of IAV infection along the neuro-immune axis, thereby most likely also affecting chronic respiratory diseases.

### 4.3. Infections with Respiratory Syncytial Virus

RSV is one of the most important respiratory viral pathogens during childhood and is associated with significant mortality. Most children will experience RSV infection by the age of 2 years. The major characteristics and symptoms are acute bronchiolitis, mucosal and submucosal edema, and luminal occlusion [116]. There is evidence that infection with RSV has effects on the local neuro-immune crosstalk and thereby may affect other respiratory diseases. SP is known to have immunomodulatory potential and specifically regulates the functions of T and B lymphocytes, monocytes, and macrophages [117,118]. Experiments in trachea, main stem bronchi, and lungs of rats showed the presence of RSV antigens on the surface and in the cytoplasm of bronchiolar epithelial cells. No virus was detected in the epithelium of more proximal airways, including the trachea and large bronchi [119]. At the same time, SP receptor (neurokinin 1 receptor) expression was upregulated by RSV [119] and such upregulation has also been associated to chronic inflammatory conditions [118]. Furthermore, it could be shown that non-neuronal cells such as lymphocytes and monocytes express SP and its receptors [120,121]. The activation of these cells that are associated with the release of cytokines like TNF-α, IL-1, IL-6, IL-8, and IL-10 [122,123], are of high interest because they can at the same time promote airway inflammation.

Altogether, next to these cytokines, especially SP may be an important contributor to the inflammatory response during RSV infection, as it is produced in the lungs of infected mice and inhibition of SP alters the inflammatory cell infiltrate and cytokine expression. 

### 4.4. Infections and Colonization with Staphylococcus aureus

*Staphylococcus aureus* (*S. aureus*) is being recognized as a major co-factor in atopic diseases like allergic asthma as it manipulates skin and mucosal immune responses at different levels [124]. Various studies suggest that exposure to *S. aureus* and its associated enterotoxins is a risk factor for the development of allergic asthma [125]. Experimentally, we recently showed that intranasally administered *S. aureus* enterotoxin B harbors a versatile potential to modulate allergic airway inflammation [126]. During *S. aureus* lung infection, nociceptors regulate protective immunity through CGRP release via the transient receptor potential ion channel 1 (TRPV1). CGRP decreases the recruitment of neutrophils that mediate killing of pathogens. Therefore, nociceptor ablation increases survival and bacterial clearance in the lung [127]. Sensory neurons that express the TRPV1 ion channel are known to mediate AHR in asthma [48], and activators of TRPV1 induce an increased cough response in COPD patients by activating neuronal TRPV1.

In infections with *S. aureus*, SP can play a likewise significant role, as it has been shown to stimulate the virulence of *S. aureus* in a reconstructed human epidermis model [128]. Since SP is also found in the lungs, one can speculate an equal or similar effect of SP upon respiratory *S. aureus* infection. Sio et al. showed that the enhanced release of SP in the lung could be a critical factor in leading to the development of systemic inflammation and pathogenesis of lung organ damage [129].

Future studies will need to elucidate to what extent neuro-immune interactions potentially contribute to the interactions between *S. aureus* and especially allergic asthma. In this context, also the neuro-immunological consequences of persistent respiratory colonization, as opposed to acute infection, will be of special interest.

## 5. Conclusions and Future Directions

We believe that we are only beginning to understand an until now underestimated role of neuro-inflammation and neuro-immune crosstalk in the interplay between respiratory infections, respiratory microbial colonization, and chronic respiratory diseases. A number of neuronal effectors such as SP and CGRP have been shown to be affected in asthma, COPD, and respiratory infection, possibly making them relevant targets for intervention. For others, there are substantial knowledge gaps, especially with respect to infection (Table 1).

**Figure 1:**
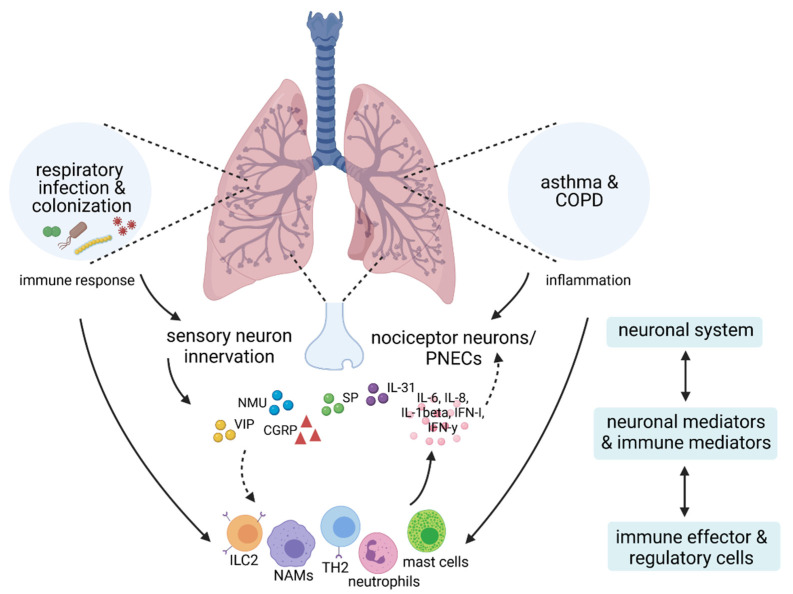
Proposed interactions between chronic respiratory diseases and respiratory pathogens along the neuro-immune axis. Schematic illustration of a proposed neuro-immunological network including cells and mediators of the neuronal and immune system that contribute to the interplay between chronic respiratory diseases and respiratory infections by direct and indirect interaction. COPD = chronic obstructive pulmonary disease, PNECs = pulmonary neuroendocrine cells, IL = interleukin, IFN = interferon, SP = substance P, VIP = vasoactive intestinal peptide, CGRP = calcitonin gene related peptide, NMU = neuromedin U, ILC2s = group 2 innate lymphoid cells, NAMs = nerve- and airway associated macrophages, TH2 = T helper type 2. Created with BioRender.com.

Based on the findings summarized in this review, we propose a neuro-immunological component to the interactions between chronic respiratory diseases and respiratory infections, as well as colonization (Figure 1). Aiming at the identification and verification of novel therapeutic targets, future clinical as well as experimental studies in combined models will have to identify the relevant disease-associated as well as pathogen-associated pathways that modulate the susceptibility to as well as the progression and exacerbation of chronic respiratory diseases. Specifically, these will on the one hand include the identification of neurological consequences of chronic respiratory diseases that affect immune responses to acute respiratory infections, exacerbating the underlying disease. On the other hand, also neuronal system-associated processes in response to infection or persistent colonization are likely to affect inflammatory pathways in chronic respiratory diseases and need to be defined. 

## Figures and Tables

**Table 1:** Effects in asthma, COPD, and respiratory infections for selected players in neuro-immune crosstalk.

	Asthma	COPD	Respiratory Infections
SP	patients are hyperresponsive to SP; SP causes bronchoconstriction and increases mucus secretion [51]. SP facilitates immune cell migration [53].	predominantly neutrophilic inflammation in COPD [87]. SP expression by neutrophils; SP enhances neutrophil phagocytic activity and regulates influx [85]. SP increases neutrophil adhesion to bronchial epithelial cells [86].	SP RNA expression is reduced in RV-infected children [99,100]. SP receptor expression is upregulated by RSV [119]. SP stimulates virulence of *S. aureus* [128].
VIP	VIP directly affects immune cells; IL-5 stimulates nociceptors to release VIP [49].	VIP reduced cigarette smoke-induced damage of alveolar cells [81]. VIP receptors are increased in the central airways of smokers with chronic bronchitis [82].	
PNECs	release of CGRP by PNECs stimulates ILC2s [74]. induce goblet-cell hyperplasia via GABA [74]. lungs from human asthmatics show increased PNECs [74].	over-activation of PNECs in COPD patients have been reported [89].	
CGRP	CGRP generates neurogenic inflammation [50]. release of CGRP stimulates ILC2s and elicits downstream responses [74].	increased sputum CGRP [89]. increased numbers of CGRP-positive cells in the airways [90].	the number of CGRP immune-reactive sensory neurons is significantly increased during IAV infection [112]. nociceptors regulate protective immunity to *S. aureus* through CGRP release [127].

COPD = chronic obstructive pulmonary disease, SP = substance P, RV = rhinovirus, RSV = respiratory syncytial virus, *S. aureus* = *Staphylococcus aureus*, VIP = vasoactive intestinal peptide, IL = interleukin, PNECs = pulmonary neuroendocrine cells, CGRP = calcitonin gene related peptide, ILC2s = group 2 innate lymphoid cells, GABA = gamma-aminobutyric acid, IAV = influenza A virus.
